# Insights into the Synthesis Mechanisms of Ag-Cu_3_P-GaP Multicomponent Nanoparticles

**DOI:** 10.1021/acsnano.3c00140

**Published:** 2023-04-05

**Authors:** Michael S. Seifner, Tianyi Hu, Markus Snellman, Daniel Jacobsson, Knut Deppert, Maria E. Messing, Kimberly A. Dick

**Affiliations:** † Centre for Analysis and Synthesis, Lund University, Box 124, 22100 Lund, Sweden; ‡ NanoLund, Lund University, Box 118, 22100 Lund, Sweden; § Solid State Physics, Lund University, Box 118, 22100 Lund, Sweden; ∥ National Center for High Resolution Electron Microscopy, Lund University, Box 124, 22100 Lund, Sweden

**Keywords:** *in situ* TEM, metal−semiconductor, heterostructures, III−V semiconductors, ion exchange, topotaxy, interfaces

## Abstract

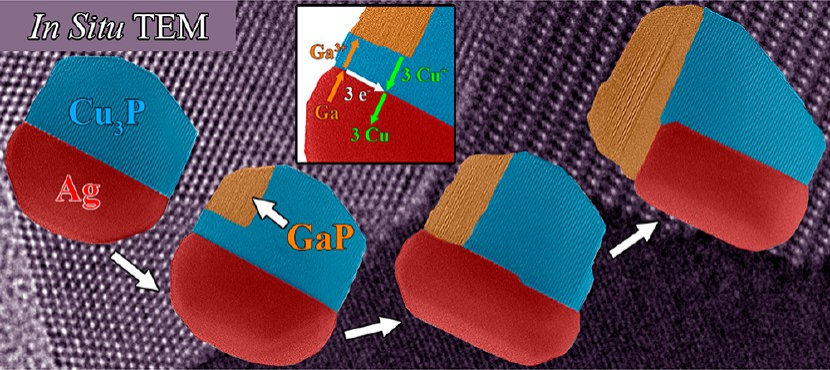

Metal–semiconductor nanoparticle heterostructures are exciting materials for photocatalytic applications. Phase and facet engineering are critical for designing highly efficient catalysts. Therefore, understanding processes occurring during the nanostructure synthesis is crucial to gain control over properties such as the surface and interface facets’ orientations, morphology, and crystal structure. However, the characterization of nanostructures after the synthesis makes clarifying their formation mechanisms nontrivial and sometimes even impossible. In this study, we used an environmental transmission electron microscope with an integrated metal–organic chemical vapor deposition system to enlighten fundamental dynamic processes during the Ag-Cu_3_P-GaP nanoparticle synthesis using Ag-Cu_3_P seed particles. Our results reveal that the GaP phase nucleated at the Cu_3_P surface, and growth proceeded via a topotactic reaction involving counter-diffusion of Cu^+^ and Ga^3+^ cations. After the initial GaP growth steps, the Ag and Cu_3_P phases formed specific interfaces with the GaP growth front. GaP growth proceeded by a similar mechanism observed for the nucleation involving the diffusion of Cu atoms through/along the Ag phase toward other regions, followed by the redeposition of Cu_3_P at a specific Cu_3_P crystal facet, not in contact with the GaP phase. The Ag phase was essential for this process by acting as a medium enabling the efficient transport of Cu atoms away from and, simultaneously, Ga atoms toward the GaP-Cu_3_P interface. This study shows that enlightening fundamental processes is critical for progress in synthesizing phase- and facet-engineered multicomponent nanoparticles with tailored properties for specific applications, including catalysis.

Multicomponent nanostructures are promising materials for several applications,^1−5^ including catalysis,^6^ photovoltaics,^7^ bioimaging,^8^ biosensing,^9^ and phototherapy.^10^ For photocatalytic applications, combining semiconductors with metals^11^ or other semiconductors^12^ can result in efficient charge carrier generation and separation, which are crucial steps in the catalytic reaction.^13^ Besides the present phases’ identities, the facets forming the multicomponent photocatalytic materials’ interfaces/surfaces are essential for their functionality.^14^ Consequently, nanostructures’ properties can be tailored through phase^15^ and facet^16^ engineering, enabling the creation of advanced materials.

Common approaches for synthesizing multicomponent photocatalysts are based on solution-based routes, usually including capping ligands.^12,14,17^ Capping ligands can block active sites at the crystal surface facets that are consequently not accessible for reactants.^18^ On the other hand, several reports identified the controlled ligand adsorption on the crystal surface facets as promising to enhance the catalysts’ performances and selectivities.^19−22^ Besides removing capping ligands by nanostructure treatment after the synthesis^23^ or capping ligand-free solution-based approaches,^24^ another strategy is synthesizing nanoparticles by gas-phase methods without applying capping ligands to obtain clean crystal surface facets.^25−27^ Those approaches (will) help evaluate the capping ligands’ impact on the catalysts’ performances by creating capping ligand-free starting points.^28^ Although controlling the faceting of catalysts is challenging without capping ligands, progress in this field is needed.^29^

Moreover, understanding the formation mechanisms of multicomponent nanostructures is crucial for gaining control over the synthesis, which enables designing phases and surface/interface facets. The formation mechanisms are usually reconstructed by characterizing the products after the synthesis. However, the indirect determination of the formation mechanisms involves the risk of misinterpreting the results or, at the very least, making interpretations more challenging.

Therefore, *in situ* approaches, including the atomic-scale visualization of nanostructures during the entire synthesis process, are essential to progress in this field.^30−34^ Moreover, catalysts’ surface alterations have been observed due to the interactions of catalysts with different environments making *in situ* characterizations crucial for understanding the actual contribution of the designed facets to the catalysts’ performances.^35−41^

During the last decades, the metal-assisted growth of semiconductor nanostructures has developed as a promising tool enabling high control over crystal properties, including crystal structure,^42−44^ composition,^45−47^ growth direction,^48−50^ morphology,^48,51,52^ surface faceting,^53−55^ doping profile,^56−58^ and defect structure.^59−61^ The seed material’s role was limited to the capability to nucleate and selectively grow the semiconductor nanocrystal.^62−64^ Few reports considered the seed material as an active component of the synthesized nanostructure.^65−68^ We propose that the metal-assisted growth approach is promising for synthesizing advanced multicomponent nanostructures. Therefore, a deeper understanding of the involved formation mechanisms is essential to exploit its full potential.

Metal phosphides’ potential as photocatalysts has been discussed in the literature,^69−71^ and heterostructures using earth-abundant metal phosphides, such as GaP-Ni_2_P^72^ and Cu_3_P-Au,^73^ have been investigated for their performances in photocatalytic water splitting. Moreover, metal cocatalysts, including Ag, can enhance catalysts’ performances by plasmon–exciton coupling.^74−76^ Therefore, this study focused on combining GaP, Cu_3_P, and Ag in a multicomponent nanostructure.

In a gas-phase synthesis approach, we enlightened the dynamic processes following the supply of Ga- and P-containing precursors to Ag-Cu_3_P nanoparticles. The experiments were performed inside an environmental transmission electron microscope (TEM) with an integrated metal–organic chemical vapor deposition (MOCVD) system. The dynamic processes were captured by high-resolution transmission electron microscopy (HRTEM) images/movies. A detailed data analysis revealed the fundamental processes involved in GaP nucleation and growth using Ag-Cu_3_P nanoparticles as seed materials.

A topotactic reaction initiated the GaP nucleation at the Cu_3_P phase’s surface, leading to rearrangement processes in the multicomponent nanoparticle. As a result, the phases connected and formed a stable GaP growth front. Counter-diffusion of Cu^+^ and Ga^3+^ cations was involved in the growth process, with the Ag phase acting as a transport medium for Ga and Cu atoms. The formation and alteration of interfaces were critical steps in synthesizing Ag-Cu_3_P-GaP nanoparticles. The presented results will allow for a deeper understanding of the fundamental mechanisms involved in synthesizing complex multicomponent nanoparticles and help progress in designing nanomaterials with applications in different fields, including catalysis.

## Results and Discussion

### Ag-Cu_3_P Nanoparticle Synthesis

**Figure 1:**
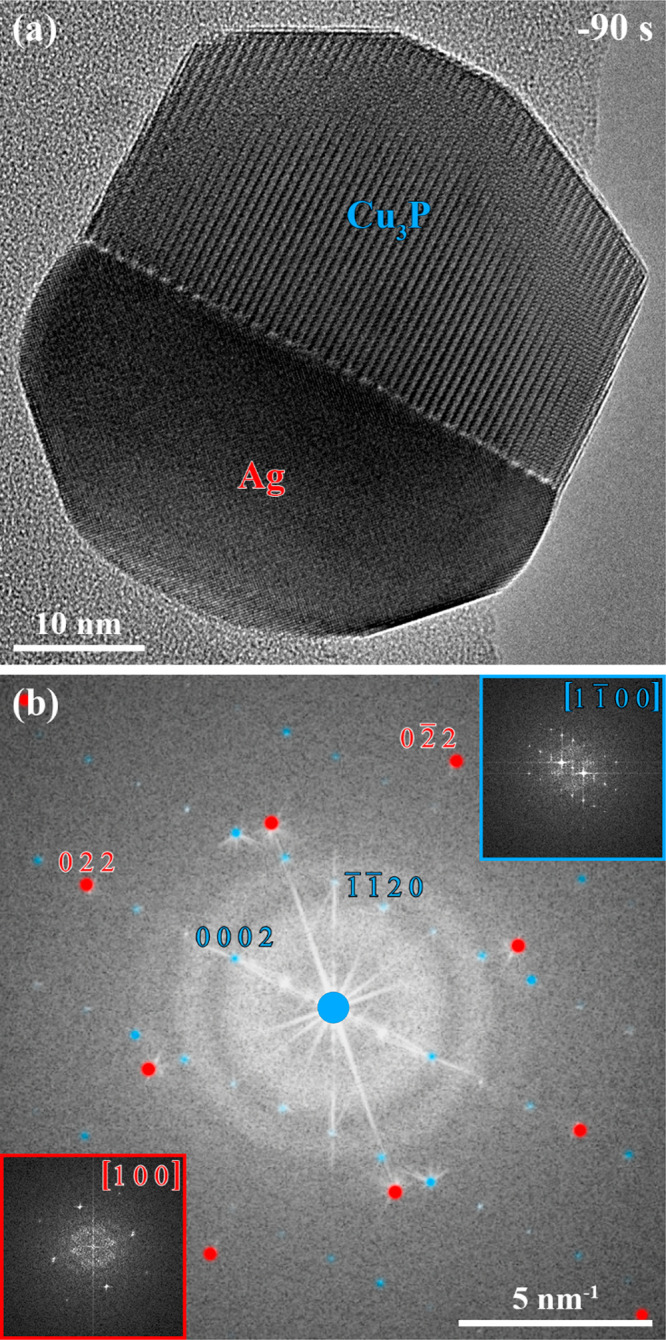
(a) The HRTEM image of an Ag-Cu_3_P nanoparticle was acquired 90 s before adding TMGa to the PH_3_ supply (timestamp: 0 s). (b) The power spectrum corresponding to the HRTEM image in (a) was overlaid with matching simulated electron diffraction patterns of the Ag (red, space group: *Fm*3*m*)^77^ and Cu_3_P (blue, space group: *P*6_3_*cm*)^78^ phases. The Ag (red, bottom left) and Cu_3_P (blue, top right) phases’ power spectra are revealed with adapted brightness and contrast as insets in (b).

The sample was prepared by depositing Ag-Cu nanoparticles with diameters of ∼30 nm on a microelectromechanical system (MEMS)-based heating chip using a home-built spark ablation system to generate and select nanoparticles with specific mobility diameters.^26^ Subsequently, the sample was transferred to the environmental TEM. Inside the environmental TEM, the sample temperature was increased to 420 °C, and phosphine (PH_3_) was supplied to initiate a selective chemical reaction with the Cu phase to form Ag-Cu_3_P nanoparticles (Figure 1).

We chose a slightly adapted Ag-Cu_3_P nanoparticle synthesis approach to the recently reported procedure by our group,^27^ excluding the pretreatment under a H_2_ atmosphere. A Ag-Cu_3_P nanoparticle with both phases oriented in one of their zone axes and the heterointerface aligned parallel to the direction of the electron beam was selected for further investigations (Figure 1a).

The HRTEM image in Figure 1a was acquired at 90 s before trimethylgallium (TMGa) was added to the precursor supply. The power spectrum corresponding to the HRTEM image in Figure 1a was overlaid with matching simulated electron diffraction patterns of the Ag (red, space group: *Fm*3*m*)^77^ and Cu_3_P (blue, space group: *P*6_3_*cm*)^78^ phases (Figure 1b). Power spectra of the phases are shown as insets in Figure 1b and allow associating the Ag and Cu_3_P phases to specific regions in the HRTEM image in Figure 1a, highlighted by color-coded labels.

It is worth mentioning that the selected Ag-Cu_3_P nanoparticle’s interface was formed by Ag{110} and Cu_3_P{1120} facets. Recently, we reported Ag-Cu nanoparticles with Ag{111}/Cu{111} interfaces acting as templates stabilizing Ag-Cu_3_P nanoparticles with interfaces formed by Ag{111} and Cu_3_P{1010} facets after initiating the chemical reaction via the supply of PH_3_.^27^ Under certain conditions, such a nanoparticle had the potential to rearrange into an Ag-Cu_3_P nanoparticle with the thermodynamically more favorable interface formed by Ag{110} and Cu_3_P{1120} facets.

The adapted synthesis procedure, lacking the pretreatment under an H_2_ atmosphere, could have led to supplying PH_3_ to Ag-Cu nanoparticles with rough heterointerfaces.^27^ That circumstance could have caused the formation of the thermodynamically more favorable Ag{110}/Cu_3_P{1120} interface in the Ag-Cu_3_P nanoparticle presented in Figure 1. However, this study does not focus on the statistical analysis of Ag-Cu_3_P nanoparticle interfaces formed by the supply of PH_3_ to Ag-Cu nanoparticles with rough heterointerfaces. Instead, this observation suggests potential approaches to further develop facet engineering in Ag-Cu_3_P nanoparticles via the presented procedure.

### Alterations in the Ag Phase Caused by Adding TMGa to the PH_3_ Supply

**Figure 2:**
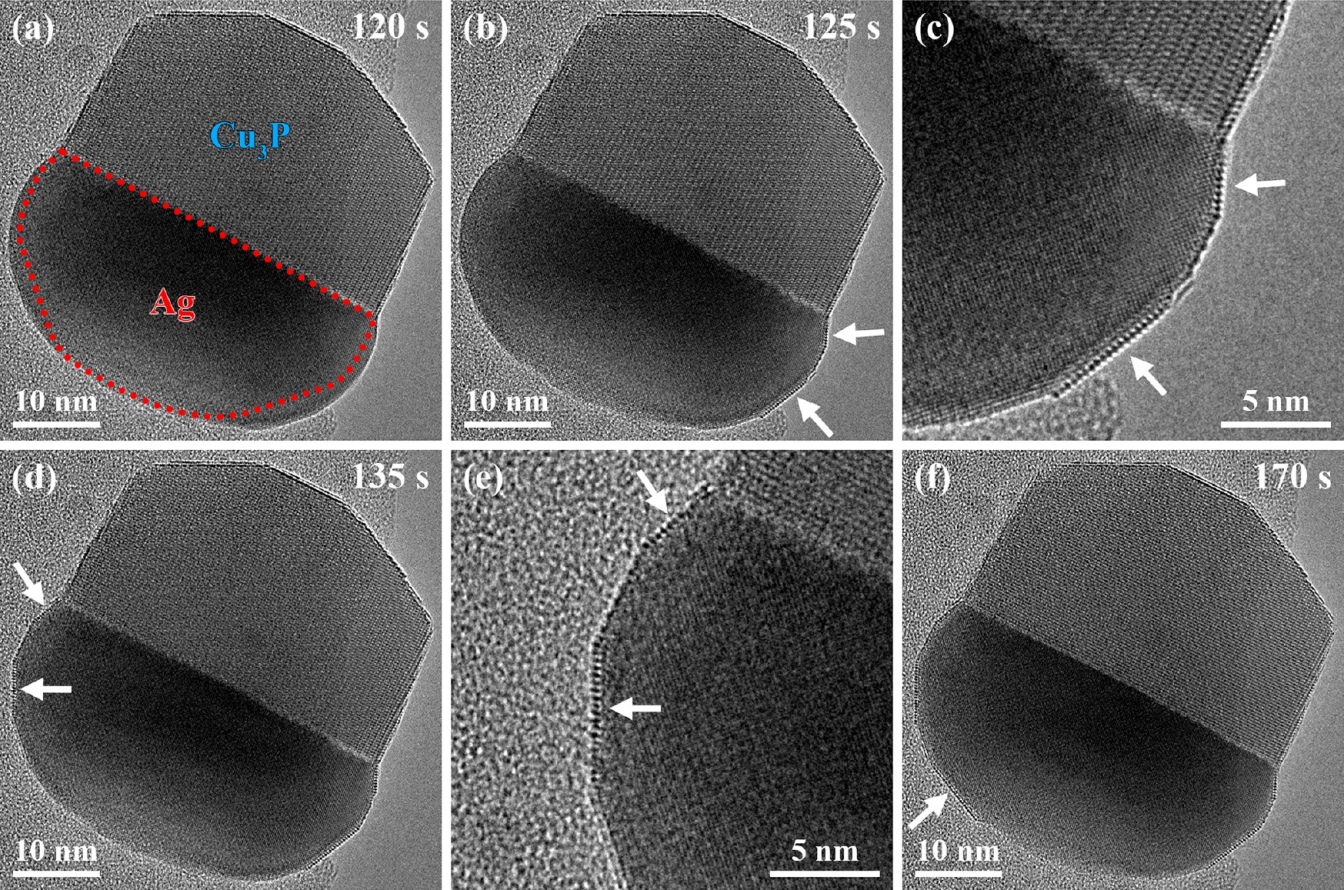
Selected averaged frames of an HRTEM movie (see Movie S1) show the Ag-Cu_3_P nanoparticle evolution in Figure 1 after adding TMGa to the precursor supply. The HRTEM image in (a) suggests that the Ag phase’s projected shape changed significantly due to the supply of Ga atoms. In contrast, the Cu_3_P phase’s projected shape remained unaffected (timestamp: 120 s). The red dotted line highlights the Ag phase’s outline before adding TMGa to the precursor supply (Figure 1, timestamp: −90 s). The white arrows in the HRTEM image in (b) indicate the faceting of the Ag phase (timestamp: 125 s). The zoomed-in region of the HRTEM image in (c) highlights the faceting of the Ag phase. The white arrows in the HRTEM image in (d) reveal the Ag phase’s faceting in other regions (timestamp: 135 s). The HRTEM image in (e) is a zoomed-in region of the HRTEM image in (d). (f) The Ag phase’s faceting continued (timestamp: 170 s), indicated by the white arrow.

Subsequently, TMGa was added to the precursor supply (timestamp: 0 s), leading to Ag-Cu_3_P nanoparticle alterations (Figure 2). The HRTEM image of the Ag-Cu_3_P nanoparticle (timestamp: 120 s) in Figure 2a was overlaid with the Ag phase’s outline (red dotted line) obtained by the HRTEM image in Figure 1a. Comparing the Ag phases’ projected shapes before and after adding TMGa to the PH_3_ supply suggests a considerable impact on the Ag phase. Although the area of the Ag phase’s projected shape increased significantly, conclusions regarding the Ag phase’s volume should be carefully drawn when working with two-dimensional projections of nanostructures. Simultaneously, no substantial changes were observed in the Ag phases’ power spectra and the Cu_3_P crystal.

Such alterations in the power spectra could be expected if Ga atoms accumulated in the Ag phase. However, a detailed analysis in Figure S1, comparing the Ag phases’ power spectra before and after adding TMGa to the PH_3_ supply, reveals no significant Ag spot alterations. Moreover, calculations show that such alterations are not expected for this specific system due to a minor decrease in the unit cell volume upon increasing the Ga concentration in the Ag phase.^79^ Therefore, the total volume of the Ag phase by adding Ga atoms to a fixed number of Ag atoms could increase without any significant alterations in the power spectrum. Based on the observed dynamic processes, the accumulation of Ga atoms in the Ag phase accompanied by increasing its total volume without significantly altering the volume of its unit cell was identified as the most probable process.

Subsequently, the Ag phase’s stepwise surface alteration was observed (Figure 2b–f). The white arrows in Figure 2b indicate the regions in the HRTEM image where the first Ag surface changes occurred (timestamp: 125 s). The surface changes were initiated at the Ag-Cu_3_P nanoparticle’s triple-phase boundary. Therefore, the zoomed-in HRTEM image of the addressed area raises the question of the type of atom/atoms that was/were located at the Ag phase’s surface (Figure 2c).

The surface alterations were also observed in other regions of the Ag phase, indicated by white arrows in Figure 2d, and started at the Ag-Cu_3_P nanoparticle’s triple-phase boundary (Figure 2d). The associated zoomed-in HRTEM image reveals that the surface atoms formed similar patterns to those observed in Figure 2c (Figure 2e). Finally, the surface alterations expanded to other regions of the Ag phase indicated by the white arrow in Figure 2f.

### GaP Nucleation

**Figure 3:**
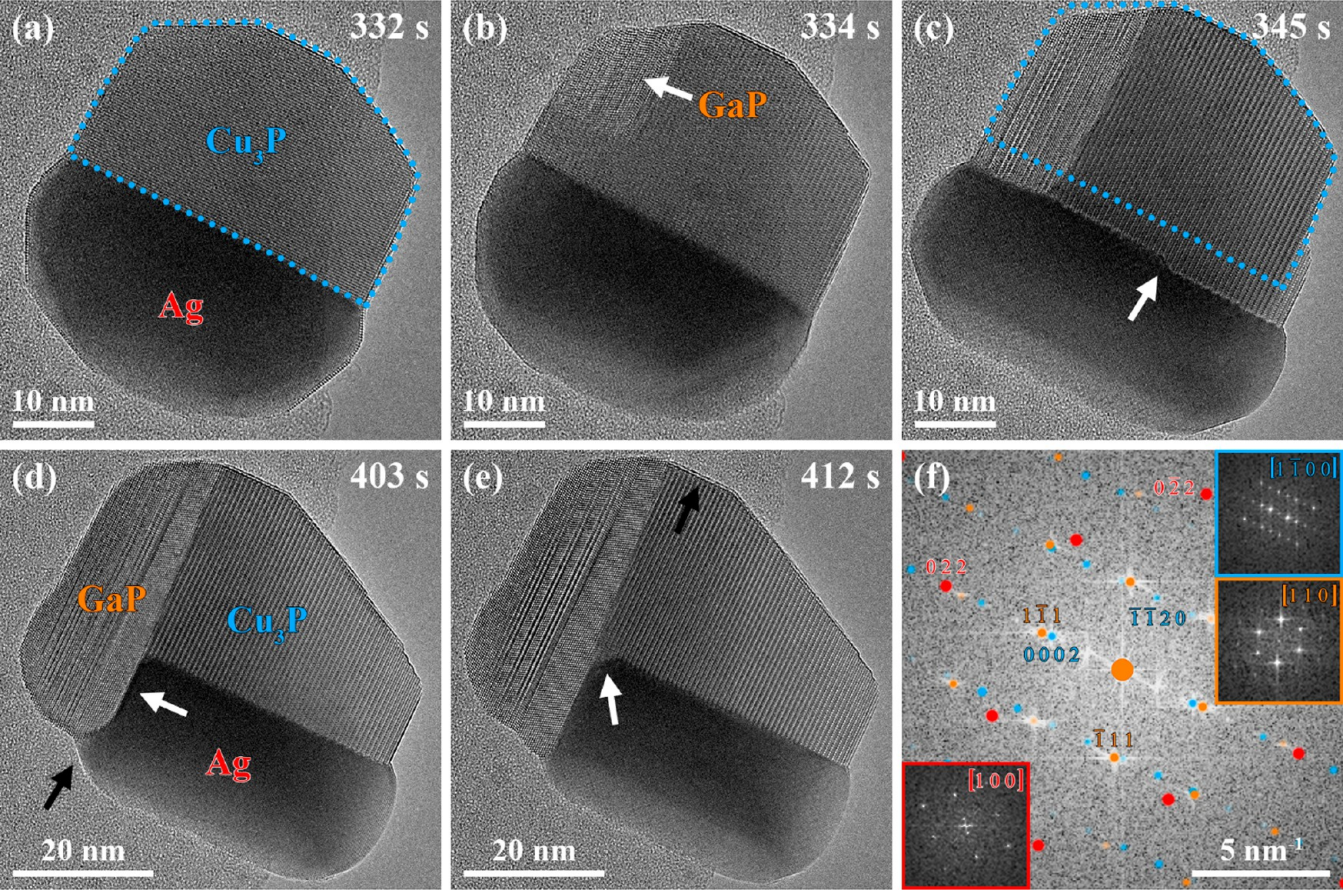
TMGa supply was increased to initiate the GaP nucleation (timestamp: 262 s). The sequence of selected averaged frames of an HRTEM movie (see Movie S2) reveals the multicomponent nanoparticle’s evolution at (a) 332 s, (b) 334 s, (c) 345 s, (d) 403 s, and (e) 412 s after adding TMGa to the precursor supply. (b) The GaP nucleation occurred at the Cu_3_P phase, and (c) the nucleus grew toward the Ag phase until all phases were in contact with each other. The blue dotted line in (c) corresponds to the outline of the Cu_3_P phase in (a). It suggests that the GaP phase’s nucleation and growth involved the removal of Cu_3_P at the GaP-Cu_3_P interface and the growth of the Cu_3_P crystal outside the area marked by the blue dotted line. The white arrow in (c) indicates an incomplete Cu_3_P layer growing while the GaP phase expanded. (d) A new GaP-Ag interface, indicated by the white arrow, formed, but the GaP growth front evolution was not completed (see region highlighted by the black arrow). (e) A stable GaP growth front formed, and an additional Ag-Cu_3_P interface appeared, indicated by the white arrow. (f) The power spectrum corresponding to the HRTEM image in (e) was overlaid with matching simulated electron diffraction patterns of the Ag (red), Cu_3_P (blue), and GaP (orange, space group: *F*43*m*)^80^ phases. The power spectra of the Ag (red, bottom left), Cu_3_P (blue, top right), and GaP (orange, center right) phases with adapted brightness and contrast are revealed as insets in (f).

The TMGa supply was subsequently increased while keeping the PH_3_ supply constant to facilitate nucleation of the GaP phase (timestamp: 262 s, Figure 3). Initially, no changes were observed in the Ag-Cu_3_P nanoparticle, with the altered Ag phase’s surface (timestamp: 332 s, Figure 3a). However, GaP nucleation occurred approximately at 72 s after increasing the TMGa supply (timestamp: 334 s, Figure 3b). The GaP nucleus formed at the Cu_3_P phase’s surface and replaced a part of the Cu_3_P crystal.

The GaP nucleus was initially not in contact with the Ag phase. However, it expanded along its [112] direction (see GaP phase’s power spectrum, Figure 3f) until the three active phases were in contact with each other (timestamp: 345 s, Figure 3c). The HRTEM image in Figure 3c was overlaid with the Cu_3_P phase’s blue dotted outline before the GaP nucleation (Figure 3a), revealing that the GaP nucleation was accompanied by the removal of Cu_3_P from the GaP-Cu_3_P interface and a growing Cu_3_P crystal in the regions outside the marked area. The white arrow in Figure 3c indicates a step in the Cu_3_P crystal moving toward the Ag-Cu_3_P-GaP triple-phase boundary as the GaP crystal expanded.

Further GaP growth led to the formation of a new interface between the Ag and GaP phases, as indicated by the white arrow in Figure 3d (timestamp: 403 s). Nevertheless, the region highlighted by the black arrow in Figure 3d reveals that the GaP growth front evolution was not completed. A stable growth front was formed at approximately 412 s after adding TMGa to the precursor flow (Figure 3e).

The power spectrum corresponding to the HRTEM image in Figure 3e was overlaid with simulated electron diffraction patterns of the Ag (red), Cu_3_P (blue), and GaP (orange, space group: *F*43*m*)^80^ phases (Figure 3f). GaP{111}/Ag{110}, GaP{111}/Cu_3_P{0001}, and Ag{110}/Cu_3_P{1120} interfaces were identified as the primary interfaces in the observed multicomponent nanostructure. Moreover, an additional Ag-Cu_3_P interface was observed directly at the triple-phase boundary between the Ag, Cu_3_P, and GaP phases.

### GaP Growth

**Figure 4:**
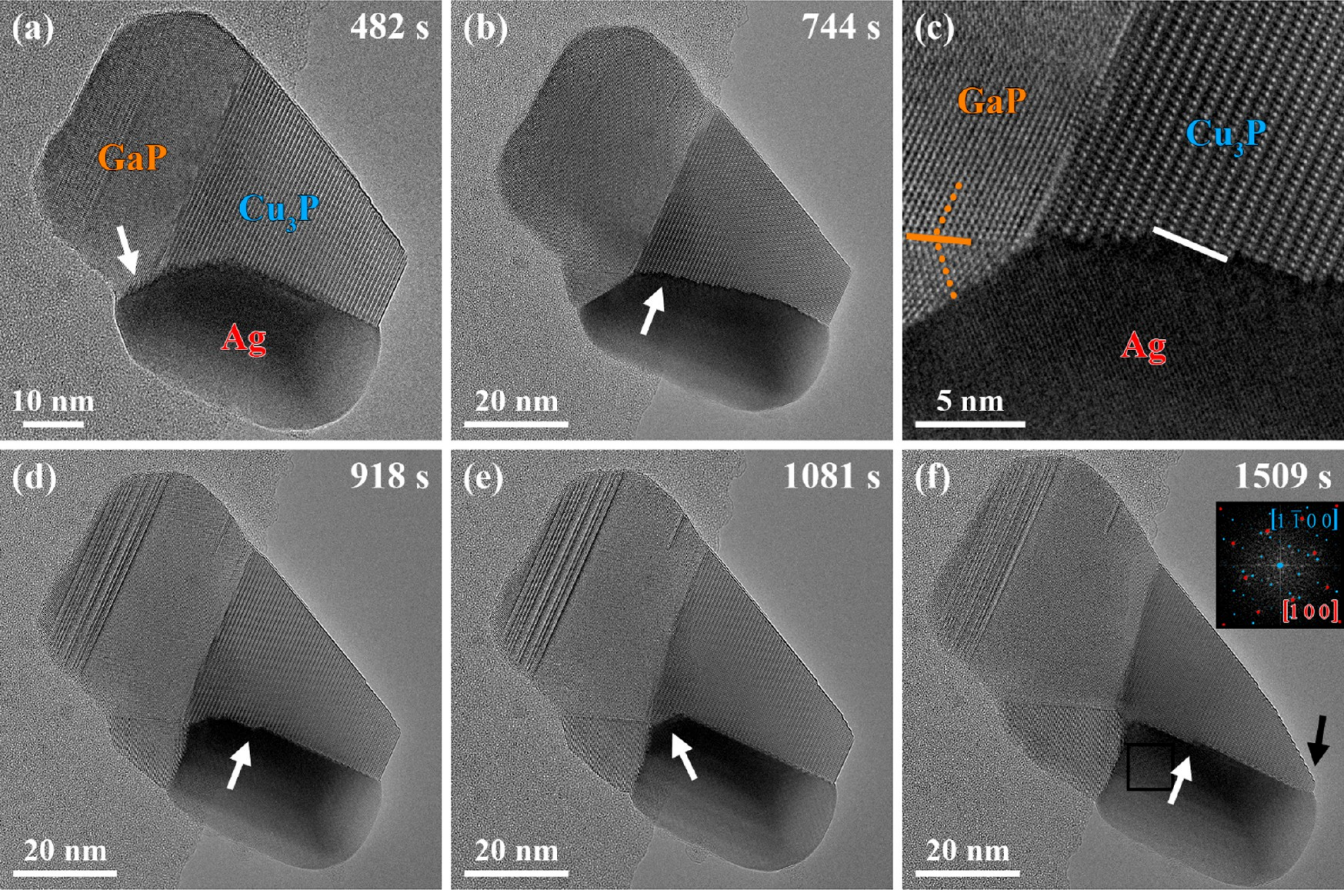
(a) The selected averaged frames of an HRTEM movie (see Movie S2) show the GaP growth front alteration indicated by the white arrow (timestamp: 482 s). The alteration was accompanied/initiated by the disappearance of the Cu_3_P crystal facet highlighted by the black arrow in Figure 3e. Subsequently, the TMGa supply was decreased while the PH_3_ supply remained constant (timestamp: 499 s) to decelerate the GaP growth, allowing detailed characterization of the observed multicomponent nanostructure. The HRTEM images were acquired at (b, c) 744 s, (d) 918 s, (e) 1081 s, and (f) 1509 s after adding TMGa to the precursor supply. (b) The HRTEM image reveals the Ag-Cu_3_P interface’s alteration, including the formation of nanofacets indicated by the white arrow. (c) The zoomed-in region of the HRTEM image in (b) allows a deeper analysis of the atomic structure at the specific heterointerfaces. Nanofacets, exemplarily indicated by the white line in (c), were observed at the Ag-Cu_3_P interface. Moreover, GaP twinning is highlighted by the mirror plane (orange line) and the mirrored GaP atomic columns (orange dotted lines). (d) The Cu_3_P crystal was removed layer-by-layer at the Ag-Cu_3_P interface, highlighted by the white arrow showing a step. (e) At the Ag-Cu_3_P-GaP triple-phase boundary, the orientation of the Cu_3_P facet deviated from the Cu_3_P facet forming the primary interface with the Ag phase. (f) The alteration of the Ag-Cu_3_P interface continued with the removal of Cu_3_P layers along the Ag-Cu_3_P interface. The step at the Ag-Cu_3_P interface highlighted by the white arrow confirms the discussed process. Since the removed Cu atoms could not attach to the shrinking top facet of the Cu_3_P crystal indicated by the black arrow, Cu_3_P growth continued in other regions. The Cu_3_P phase’s expansion already observed in (b) became more pronounced, leading to overlaps of the Ag and GaP phases with the Cu_3_P phase. The power spectrum of the area marked by the black rectangle in the HRTEM image (adapted brightness and contrast) was overlaid with matching simulated electron diffraction patterns of the Ag and Cu_3_P phases and is shown as an inset in (f). Moreover, additional reflections were observed but could not be allocated to a specific phase.

As the GaP crystal growth continued along its [111] direction, the Cu_3_P facet indicated by the black arrow in Figure 3e disappeared stepwise, and the additional Ag-Cu_3_P interface’s area increased, initiating phase rearrangements at the growth front (Figure 4). The Ag phase formed a differently oriented interface with the GaP crystal, indicated by the white arrow in Figure 4a (timestamp: 482 s). GaP growth continued at the two differently oriented GaP growth fronts, and nanofacets appeared at the Ag-Cu_3_P interface (timestamp: 744 s, Figure 4b).

The zoomed-in area of the HRTEM image indicated by the white arrow in Figure 4b reveals the atomic structure at the specific heterointerfaces (Figure 4c). While the GaP-Ag interface changed, the GaP-Cu_3_P interface formed by GaP{111} and Cu_3_P{0001} facets remained unaltered. A closer look at the GaP-Ag interface change reveals that a GaP twin was formed as part of the phase rearrangements. The orange line indicates the twin’s mirror plane, and the orange dotted lines highlight the mirrored GaP atomic columns in Figure 4c.

Moreover, the Ag-Cu_3_P interface changed significantly by developing local Ag{110}/Cu_3_P{1120} interfaces, exemplarily highlighted by the white line in Figure 4c. The local Ag-Cu_3_P interfaces were interrupted by crystal steps. This observation could have been the direct consequence of the GaP twin formation leading to a slight rotation of the Ag phase relative to the Cu_3_P phase. Consequently, the steps at the Ag-Cu_3_P interface could hint toward dislocations accommodating the strain originating from the phases’ mismatch already observed in a previous study.^27^

It is worth mentioning that a moiré pattern was visible in the Ag phase in Figure 4b due to an overlap of the Ag and Cu_3_P phases. The moiré pattern expanded from the Ag-Cu_3_P interface over a larger area (timestamp: 918 s, Figure 4d). This observation suggests that the phases formed additional heterointerfaces with differently oriented facets. Moreover, the GaP-Ag interface continuously changed and was no longer oriented parallel to the direction of the electron beam. Nevertheless, the addressed processes led to the accommodation of strain indicated by the continuous removal of crystal steps at the Ag-Cu_3_P interface highlighted by the white arrow in Figure 4d.

After the removal of the crystal steps at the Ag-Cu_3_P interface, again, an additional Ag-Cu_3_P interface indicated by the white arrow in Figure 4e remained beside the primary Ag{110}/Cu_3_P{1120} interface (timestamp: 1081 s). The white arrow in Figure 4f reveals the removal of Cu_3_P along the Cu_3_P[0002] direction via a moving crystal step at the Ag{110}/Cu_3_P{1120} interface. The Cu atoms diffused toward the top facet of the Cu_3_P crystal (see black arrow, Figure 4f) and reacted with the supplied P atoms to form Cu_3_P layers. The area of the Cu_3_P crystal’s top facet decreased during this process. Therefore, other available Cu_3_P crystal facets grew, leading to the moiré patterns observed in Figure 4e,f.

The power spectrum of the HRTEM image region marked by the black rectangle in Figure 4f was overlaid with matching simulated electron diffraction patterns of the Ag and Cu_3_P phases (see inset in Figure 4f). These results confirm the growth of the Cu_3_P crystal in regions other than the addressed top facet. Moreover, additional reflections that could not be associated with a specific phase were observed. Since the Cu_3_P phase was in contact with the twinned GaP segment, the additional reflections likely corresponded to a differently oriented Cu_3_P crystal. A detailed analysis of regions with overlapping crystals causing moiré patterns can be found in Figure S2.

### Ag Phase’s Surface Alterations

**Figure 5:**
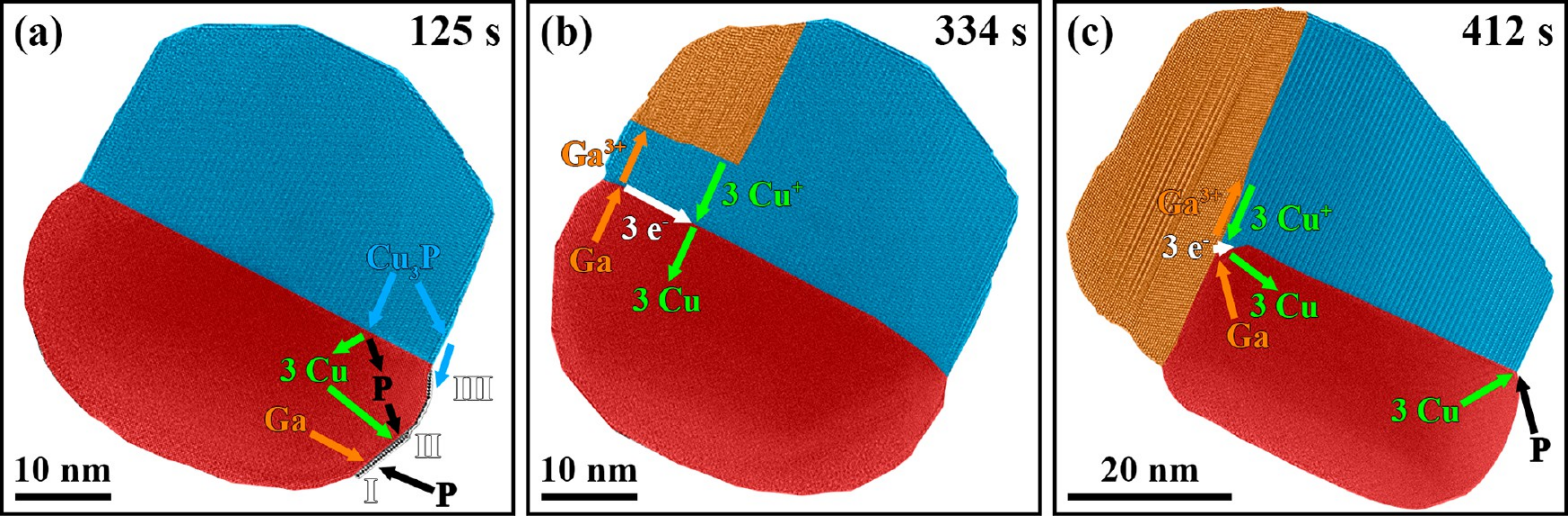
Schematic representations of (a) Figure 2b, (b) Figure 3b, and (c) Figure 3e are shown to illustrate the nucleation and growth mechanisms as well as the underlying potential diffusion pathways. (a) The reaction of Ga atoms with supplied P atoms (mechanism I), the Ag-mediated dissolution and redeposition of Cu_3_P (mechanism II), and the surface diffusion of Cu and P atoms across the Ag-Cu_3_P interface (mechanism III) could have caused the observed Ag phase’s surface alterations. (b) The GaP nucleation and growth required exchanging Cu^+^ and Ga^3+^ cations through the Cu_3_P phase and a redox reaction at the Ag-Cu_3_P interface. (c) After forming a stable GaP growth front, a similar process was observed involving the exchange of Cu^+^ and Ga^3+^ cations along the GaP-Cu_3_P interface. Moreover, Cu_3_P was redeposited at a thermodynamically favorable Cu_3_P facet in contact with the Ag and gas phases. The Ag phase acted as a diffusion medium for the Cu atoms, and the P atoms supplied via the gas phase led to a chemical reaction at the Ag-Cu_3_P-gas triple-phase boundary.

Now, the dynamic processes observed during the experiment and discussed above will be used to enlighten the Ag phase’s surface alterations and the mechanisms of GaP nucleation and growth at an atomic scale (Figure 5). Changes in the Ag phase’s surface were observed after adding TMGa to the PH_3_ supply. Based on our observations, we conclude that Ga accumulation in the Ag phase was the most probable initial step causing the formation of surface layers. However, since we could not directly prove the enrichment of the Ag phase with Ga atoms by complementary methods, alternative scenarios leading to this observation are added to the discussion (Figure 5a).

In the first scenario, the Ag phase’s oversaturation with Ga atoms could have led to the diffusion of Ga atoms toward the Ag phase’s surface and a subsequent chemical reaction with P atoms supplied by the gas phase (mechanism I, Figure 5a). In the second scenario, the Ag phase could have mediated the Cu_3_P phase’s partial dissolution at the Ag-Cu_3_P interface. Subsequently, the Cu_3_P compound could have been redeposited at the Ag phase’s surface (mechanism II, Figure 5a). In the third scenario, Cu and P atoms could have diffused along the surface across the heterointerface and covered the Ag phase’s surface (mechanism III, Figure 5a).

In the literature, ordered layers were observed at liquid–solid,^81^ liquid–vapor,^82^ and solid–vapor^40^ interfaces. The formation mechanisms included the supersaturation of a phase by specific atomic species segregating at the phase’s surface^82^ and the surface diffusion of atomic species from one phase to another.^40^ However, it is worth mentioning that the methods used in this study did not allow the clarification of the exact formation mechanism.

Consequently, complementary methods are essential for studying dynamic processes at an atomic scale. One possible (but challenging) way to gain a deeper understanding of the altered Ag phase’s surface might be the determination of its chemical composition using high-resolution scanning transmission electron microscopy (STEM)-electron energy loss spectroscopy (EELS). Moreover, complementary theoretical modeling could be used to compare the suggested formation mechanisms and determine the likelihood of their occurrences.^83^

### Nucleation Mechanism

In contrast to the above case, a more detailed analysis of the diffusion processes was possible for the GaP nucleation. The combination of observations, including the GaP nucleus formation at the Cu_3_P phase’s surface, its growth toward the Ag-Cu_3_P interface by replacing the Cu_3_P phase, and alterations in the Ag phase upon adding TMGa to the PH_3_ supply, suggests that counter-diffusion of Cu^+^ and Ga^3+^ cations and redox reactions were involved in the GaP nucleation and growth (Figure 5b).

The supply of atomic species from specific regions within the multicomponent nanoparticle (e.g., P atoms from the Cu_3_P phase) and the propagation of the GaP growth front indicate the following chemical reaction as the driving force for the observed diffusion processes:
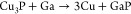
1

Therefore, Cu^+^ ions must have diffused from the GaP-Cu_3_P interface toward the Ag-Cu_3_P interface. At the Ag-Cu_3_P interface, the Ga atoms supplied directly by the Ag phase or through diffusion along the Ag-Cu_3_P interface reduced the Cu^+^ cations by being oxidized to Ga^3+^ cations. Simultaneously, the resulting Ga^3+^ cations diffused toward the GaP-Cu_3_P interface, reacting with the P^3–^ anions of the Cu_3_P phase’s anion sublattice. The Cu_3_P phase’s crystal structure was essential to facilitate this process and matched well with the GaP phase’s crystal structure (see Figure S3). Consequently, the discussed process can be described as a topotactic reaction.

A similar observation was reported in the literature showing the (partial) replacement of the Cu_3_P phase by GaP via a cation exchange reaction in a solution-based process.^84^ The high concentration of Cu vacancies in Cu_3_P nanocrystals was identified as essential for their capability to perform cation exchange reactions.^85^ The same approach, using Cu_3_P nanocrystals as starting materials, was applied to synthesize InP^84−87^ and In_1–*x*_Ga_*x*_P^84^ nanoparticles. Here, instead of a solution containing Ga^3+^ cations, the Ag phase mediated the supply of Ga atoms.

The above-mentioned studies reveal the stabilization of III–V compounds, including GaP,^84^ in their metastable wurtzite polytype. In this study, GaP nuclei with the zincblende crystal structure were synthesized. The formation of stacking faults in the GaP nucleus (see Figure 3b) could have been caused by the slightly different formation mechanism and a potential minor mismatch between the phases at the heterointerface (see Figure S3). After the Ag and Cu_3_P phases were rearranged at the GaP growth front, changing the growth mechanism (see Figure 3d), no stacking faults were observed in the associated GaP segment.

Consequently, the choice of synthesis parameters, such as the temperature, could impact the stabilization of specific III–V semiconductor polytypes. Moreover, it is worth mentioning that literature reported the presence of interfaces consisting of Cu_3_P{1120} and InP{1100} facets when Cu_3_P crystals were partly replaced by wurtzite InP segments in cation exchange reactions.^85^ Although, in the here-presented study, the observed GaP-Cu_3_P growth front also involved a Cu_3_P{1120} facet during the GaP nucleation, another GaP-Cu_3_P interface involving a Cu_3_P{0001} facet formed (see Figure 3b). This additional heterointerface could have prevented stabilizing the metastable wurtzite polytype. Therefore, controlling the heterointerfaces evolving during the nucleation step by synthesizing defined Ag-Cu_3_P seed particles could be essential for crystal structure engineering via the here-presented approach.

Finally, it is worth mentioning that the observed dynamic processes and formation mechanisms correspond to a specific set of synthesis parameters. Therefore, temperature, partial pressure, and carrier gas variations will likely result in alternative synthesis mechanisms. In particular, under certain conditions, the Ag phase could initiate GaP nucleation at the Ag-Cu_3_P-gas triple-phase boundary, similar to the reported Ag-assisted nucleation of GaP nanowires on a substrate.^88^

### Growth Mechanism

The growth mechanism with associated diffusion pathways could also be clarified after the initial GaP nucleation step (Figure 5c). In particular, similar to the GaP nucleation discussed above, the chemical reaction 1 was indirectly observed at the Ag-Cu_3_P-GaP triple-phase boundary.

The stepwise replacement of Cu_3_P by GaP evolved from the Ag-Cu_3_P-GaP triple-phase boundary. It led to crystal steps at the GaP-Cu_3_P interface since Cu^+^ and Ga^3+^ cations had to be interchanged along the GaP-Cu_3_P interface, requiring more time for Cu^+^ cations farther away from the triple-phase boundary (see Figure 3e). Simultaneously, the Cu^+^ cations that were reduced to Cu atoms at the triple-phase boundary diffused through/along the Ag phase to a thermodynamically favorable Cu_3_P facet at the Ag-Cu_3_P-gas triple-phase boundary. The Cu atoms then reacted with the supplied P atoms to form Cu_3_P, which was redeposited at the addressed Cu_3_P facet.

The growth mechanism and diffusion pathways became more complex after the Cu_3_P facet, indicated by the black arrow in Figure 3e, disappeared, and a GaP twin was observed. Likely, the twinning of the GaP phase led to the formation of a differently oriented Cu_3_P phase segment, which made the analysis nontrivial. Nevertheless, it can be expected that after the GaP twinning, the chemical reaction 1 and the redeposition of Cu_3_P mediated by the Ag phase were essential steps to facilitate the GaP growth.

## Conclusions

The results herein enlighten the fundamental processes involved in synthesizing Ag-Cu_3_P-GaP nanoparticles by supplying Ga- and P-containing precursors to Ag-Cu_3_P seed particles. Straight after supplying both precursors, dynamic processes were observed solely in the Ag phase, including the formation of ordered layers at the Ag phase’s surface.

Subsequently, GaP nucleated at the Cu_3_P phase’s surface and grew toward the Ag-Cu_3_P interface. We identified the counter-diffusion of Cu^+^ and Ga^3+^ cations through the Cu_3_P phase, facilitated by the high Cu vacancy concentration in the Cu_3_P phase, as an essential process enabling GaP nucleation. After the Ag and Cu_3_P phases were rearranged at the GaP growth front, similar mechanisms were involved in promoting GaP growth at the GaP-Cu_3_P interface. Cu atoms accumulated in/at the Ag phase and diffused to the Ag-Cu_3_P-gas triple-phase boundary where the Cu atoms reacted with supplied P atoms forming Cu_3_P at a specific Cu_3_P crystal facet, not in contact with the GaP phase.

After the addressed Cu_3_P crystal facet’s area decreased significantly, Cu_3_P growth continued in other regions, indicated by moiré patterns, and additional interfaces between the phases were formed. Consequently, further GaP growth became complex and involved significant Ag-Cu_3_P interface reconstructions. However, despite the slightly different mechanisms for GaP nucleation and growth, the Cu_3_P acting as a P reservoir and the Ag phase mediating the diffusion of Ga and Cu atoms were essential for synthesizing Ag-Cu_3_P-GaP multicomponent nanoparticles.

This study enlightens the fundamental formation mechanisms of multicomponent nanoparticles. Epitaxial relationships, diffusion of cations/atoms, and redox reactions at interfaces have been identified as essential aspects of the synthesis that need to be considered when aiming to control multicomponent nanoparticles’ properties. The findings of this study will help create interface facet-engineered multicomponent nanoparticles with potential in catalysis.

## Methods

### Ag-Cu Nanoparticle Synthesis and Deposition on MEMS-Based Heating Chips

Ag-Cu agglomerates were generated in a home-built spark ablation system.^89^ In the reactor, a spark between a Ag anode and a Cu cathode ablated material from both electrodes. A N_2_/H_2_ carrier gas was used to transport the formed agglomerates from the reactor through a furnace kept at 850 °C to sinter the agglomerates into spherical, phase-segregated nanoparticles. A differential mobility analyzer system allowed sintered bimetallic nanoparticles with a selected mobility diameter of 30 nm to remain in the gas stream. The selected bimetallic nanoparticles were deposited on a MEMS-based heating chip by an electrostatic precipitator. A detailed process description, including relevant parameters, can be found in a recently published study.^26^

### MEMS-Based Heating Chips for *in Situ* TEM Investigations

MEMS-based chips with a thin SiN_*x*_ membrane and an embedded W coil for Joule heating from Norcada Inc. were used for the *in situ* TEM experiments. The SiN_*x*_ membrane contained 19 regions with a few nm thick SiN_*x*_ layer and holes in their center. The chips allowed sample heating up to 1100 °C with a homogeneous temperature profile along the central region of the chip. The temperature variation was performed in constant resistance mode and controlled by the Blaze software supplied by Hitachi High-Technologies.

### Environmental TEM and HRTEM Image/Movie Acquisition

A Hitachi HF-3300S environmental TEM operated at 300 kV was used to perform the experiments. The microscope was equipped with a cold field emission gun as the electron source, an imaging aberration corrector (CEOS BCOR), and a complementary metal–oxide–semiconductor (CMOS) camera (Gatan OneView IS camera). Electron dose rates of ∼1500 e/Å^2^ s to 4400 e/Å^2^ s were used to acquire HRTEM images and movies (see Tables S1 and S2). The background pressure next to the sample was ∼9.20 × 10^–4^ Pa. Detailed information about the used environmental TEM and its capabilities are available elsewhere.^90^

### Sample Holder and Precursor Supply

A custom-built double tilt holder from Hitachi was used to perform the experiments. The gas handling system enabled the controlled supply of PH_3_ and TMGa via a setup similar to the ones used in commercially available MOCVD systems. It is worth mentioning that PH_3_ was supplied in its pure form. In contrast, TMGa was stored in a bubbler kept at −20 °C. Its condensed phase was in equilibrium with the gas phase, which was transported from the bubbler via the H_2_ carrier gas to the gas handling system. The TMGa/H_2_ mixture was further diluted with H_2_ before entering the microscope. The precursor flows were supplied directly to the sample via separate lines of a side port injector integrated into the microscope column. The chemicals used in this study, including PH_3_ (flammable, toxic) and TMGa (pyrophoric), must be handled with care and appropriate equipment.

### Process Parameters for the Experiment

The first step of the approach to nucleate GaP using bimetallic seed particles was forming Ag-Cu_3_P nanoparticles. The Ag-Cu nanoparticles were not pretreated under an H_2_ atmosphere at elevated temperatures, as previously reported by our group.^27^ The adapted procedure is summarized in Table S3. The GaP nucleation was initiated at a temperature of 420 °C by adding TMGa to the PH_3_ supply. The TMGa supply was increased stepwise to achieve GaP nucleation. After the nucleation event, the TMGa supply was decreased significantly to reduce the GaP growth rate. The PH_3_ supply was kept constant during the whole experiment. The process parameters used for the GaP nucleation and growth by Ag-Cu_3_P seed particles are summarized in Tables S4 and S5. The total and partial pressures were estimated based on a recently published study.^90^ The graph in Figure S4 reveals the variation of process conditions over time and specific experiment events.

### Simulation of Electron Diffraction Patterns

Power spectra were loaded in the SingleCrystal software, and the corresponding scale bars were used to set the camera lengths for the electron diffraction pattern simulations. The cif files were downloaded from the Inorganic Crystal Structure Database. The parameters for the SingleCrystal simulations are summarized in Table S6. The simulations did not consider the phases’ thermal expansions due to the lack of the Cu_3_P phase’s thermal expansion coefficient in the literature. It is worth mentioning that electron diffraction patterns do not contain exactly the same information as HRTEM images’ power spectra. Still, overlaying them is an adequate method to confirm the presence of specific phases.

### Data Processing/Software

DigitalMicrograph from Gatan (version 3.50.3584.0) with the integrated *in situ* player was used to acquire and process HRTEM images and movies. Tables S7 and S8 summarize the processing parameters for extracting HRTEM images/movies from the HRTEM movie raw data. HRTEM movies in the Supporting Information were compressed and labeled (scale bars and timestamps) using ImageJ (version 1.53p). The power spectra were calculated using DigitalMicrograph and loaded in SingleCrystal from CrystalMaker Software Ltd. (version 4.1.6) to overlay them with simulated electron diffraction patterns. The figures were prepared using Adobe Photoshop from Adobe (version 22.1.0).

## Supporting Information Available

Figures S1–S4 show a comparison between the Ag phases’ power spectra before and after adding TMGa to the PH_3_ supply, an analysis of the observed moiré patterns, the crystal structure matching between the GaP and Cu_3_P phases at their interface, and a graph that reveals the variation of process conditions over time and specific experiment events; Tables S1–S8 contain information about the acquisition parameters for the HRTEM images/movies, the process parameters for the formation of Ag-Cu_3_P nanoparticles by Ag-Cu seed particles, the process parameters for the GaP nucleation and growth by Ag-Cu_3_P seed particles, the parameters for the electron diffraction pattern simulations, and the processing parameters for extracting HRTEM images/movies from the HRTEM movie raw data (PDF)
Movies S1: Ag phase’s surface alterations after adding TMGa to the PH_3_ supply (AVI)
Movie S2: GaP nucleation and growth after increasing the TMGa supply (AVI)

## Supplementary Material

nn3c00140_si_001.pdf

nn3c00140_si_002.avi

nn3c00140_si_003.avi

## ABBREVIATIONS

TEM: transmission electron microscope
MOCVD: metal–organic chemical vapor deposition
HRTEM: high-resolution transmission electron microscopy
microelectromechanical systems: MEMS
TMGa: trimethylgallium
STEM: scanning transmission electron microscopy
EELS: electron energy loss spectroscopy
CMOS: complementary metal–oxide–semiconductor
