# Supplementary material for: Insights into the Synthesis Mechanisms of Ag-Cu3P-GaP Multicomponent Nanoparticles
Source: ACS Nano. 2023 Apr 5;17(8):7674–84. doi: 10.1021/acsnano.3c00140 (PMC10134500; doi:10.1021/acsnano.3c00140)
Supplement: Supplementary file 1 — nn3c00140_si_001.pdf [file nn3c00140_si_001.pdf]

Supporting Information for

Insights into the Synthesis Mechanisms of Ag-Cu_3P-GaP Multicomponent Nanoparticles

Michael S. Seifner,[*,†,‡], Tianyi Hu,[†,‡] Markus Snellman,[‡,§] Daniel Jacobsson,[†,‡,∥] Knut Deppert,[‡,§] Maria E. Messing,[‡,§] and Kimberly A. Dick[*,†,‡]

[†] Centre for Analysis and Synthesis, Lund University, Box 124, 22100 Lund, Sweden

[‡] NanoLund, Lund University, Box 118, 22100 Lund, Sweden

[§] Solid State Physics, Lund University, Box 118, 22100 Lund, Sweden

[∥] National Center for High Resolution Electron Microscopy, Lund University, Box 124, 22100 Lund, Sweden

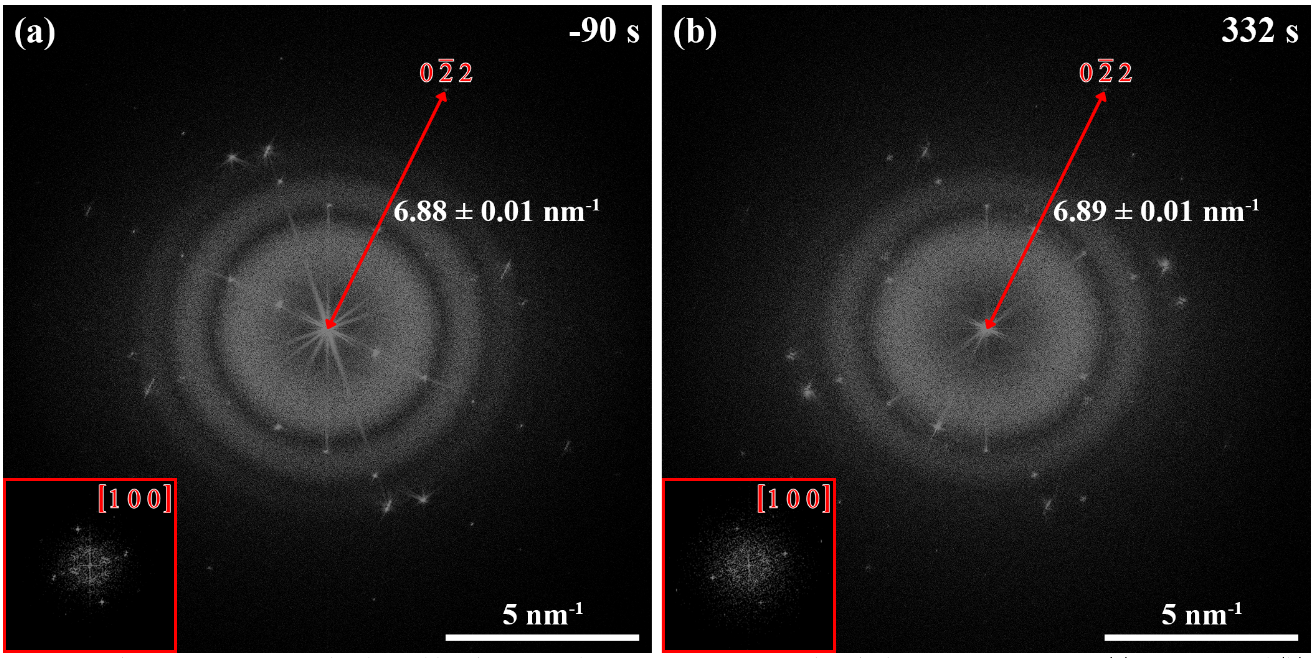

Figure S1. The power spectra with adapted brightness and contrast are associated with the HRTEM images in (a) Figure 1a and (b) Figure 3a. The Ag(0$\bar{2}$2) spots were chosen for measuring potential changes in the Ag phase's unit cells after adding TMGa to the PH_3 supply. The selected Ag spots were visible in both power spectra (see the Ag phases' power spectra shown as insets) and farthest away from the central spot. Consequently, potential variations due to unit cell changes would be more significant for those spots than other Ag spots closer to the central spot. Literature reports a decrease of the Ag phase's lattice constant by ~0.12 % upon alloying with 7.3 atom % Ga.[1] Another study suggests that this trend is maintained at higher sample temperatures such as the one used in this work (420 °C).[2] For the Ag(0$\bar{2}$2) spot, the addressed lattice parameter decrease would increase its inverse distance from the central spot from 6.88 nm^{-1} to 6.89 nm^{-1}. However, the expected change is within the error of the measurements (±0.01 nm^{-1}), making it impossible to determine the potential accumulation of Ga atoms in the Ag phase via this method. Nevertheless, substituting Ag atoms at regular lattice positions with the supplied Ga atoms would increase the number of unit cells and, therefore, (over)compensate for the volume decrease caused by the slight reduction of the Ag phase's lattice parameter.

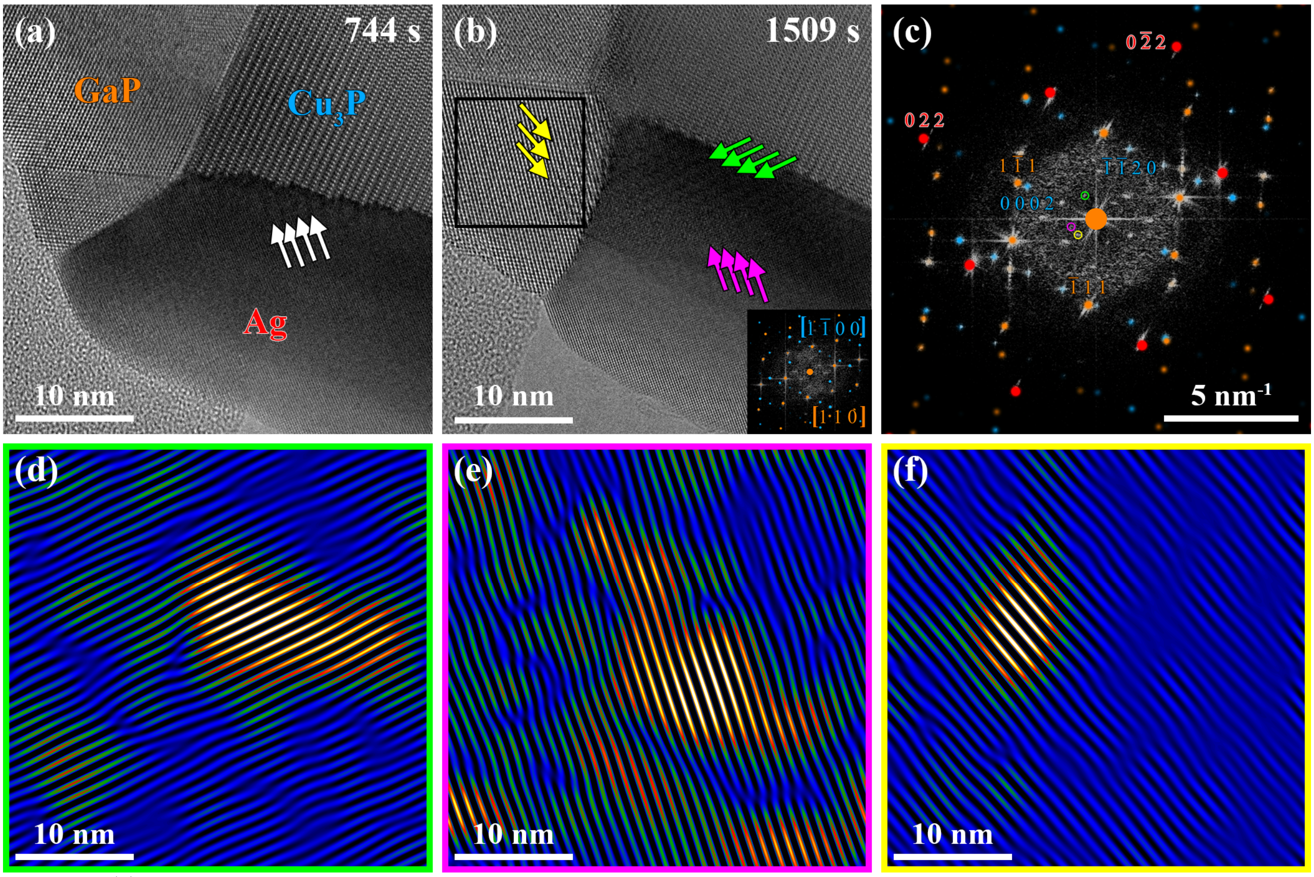

Figure S2. (a) The zoomed-in region of the HRTEM image in Figure 4b reveals an area, indicated by white arrows, where a moiré pattern evolved from the Ag-Cu_3P interface toward the Ag phase due to an overlap of the Ag and Cu_3P phases. (b) At a later stage, the zoomed-in region of the HRTEM image in Figure 4f showed more pronounced overlaps of the Ag and GaP phases with the Cu_3P phase. The phase overlaps resulted in moiré patterns indicated by green, purple, and yellow arrows. (c) The power spectrum (adapted brightness and contrast) corresponding to the HRTEM image in (b) was overlaid with simulated electron diffraction patterns of the Ag, Cu_3P, and GaP phases. The green, purple, and yellow circles indicate low spatial frequencies corresponding to the moiré patterns highlighted in (b). (d-f) The regions associated with those phase overlaps (color-coded) could be visualized by applying masks on the low spatial frequencies and, subsequently, an inverse fast Fourier transform (FFT). The overlap of the Ag and Cu_3P phases caused the low spatial frequencies visualized in (d-e). The inset in Figure 4f confirms the presence of those phases in the addressed region. In contrast, the low spatial frequencies visualized in (f) appeared due to an overlap of the GaP and Cu_3P phases. The power spectrum (adapted brightness and contrast) corresponding to the region indicated by a black rectangle in (b) was overlaid with simulated electron diffraction patterns of the GaP and Cu_3P phases, confirming their presence in the addressed region (see inset in (b)). The intensity range of the images in (d-f) varies from dark blue (low intensity) to yellow (high intensity).

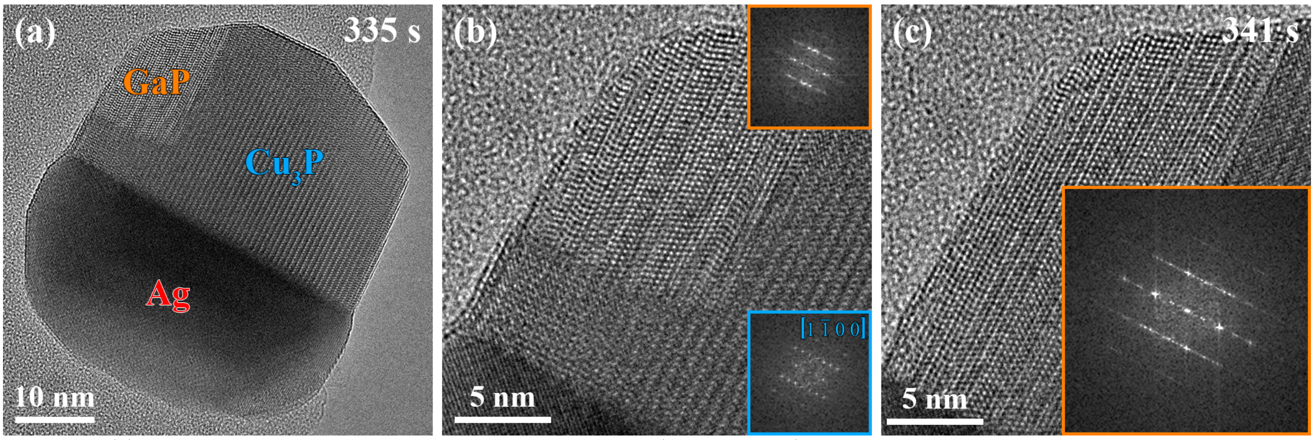

Figure S3. (a) The selected averaged frames of an HRTEM movie (see Movie S2) shows the multicomponent heterostructure in an early stage of the GaP nucleation (timestamp: 335 s). (b) The zoomed-in region of the HRTEM image in (a) and the GaP and Cu_3P phases' power spectra as insets reveal the matching of both phases' crystal structures. (c) The zoomed-in region of the selected averaged frames of an HRTEM movie (see Movie S2) highlights the same region as in (b) at a later stage of the GaP nucleation (timestamp: 341 s). The initial GaP crystal was enriched with stacking faults, likely due to the diffusion pathways involved in its formation and a slight mismatch of the GaP and Cu_3P crystals at the interface. Consequently, due to the slight lattice mismatch, the stacking faults could have been formed to accommodate strain at the GaP-Cu_3P interface.

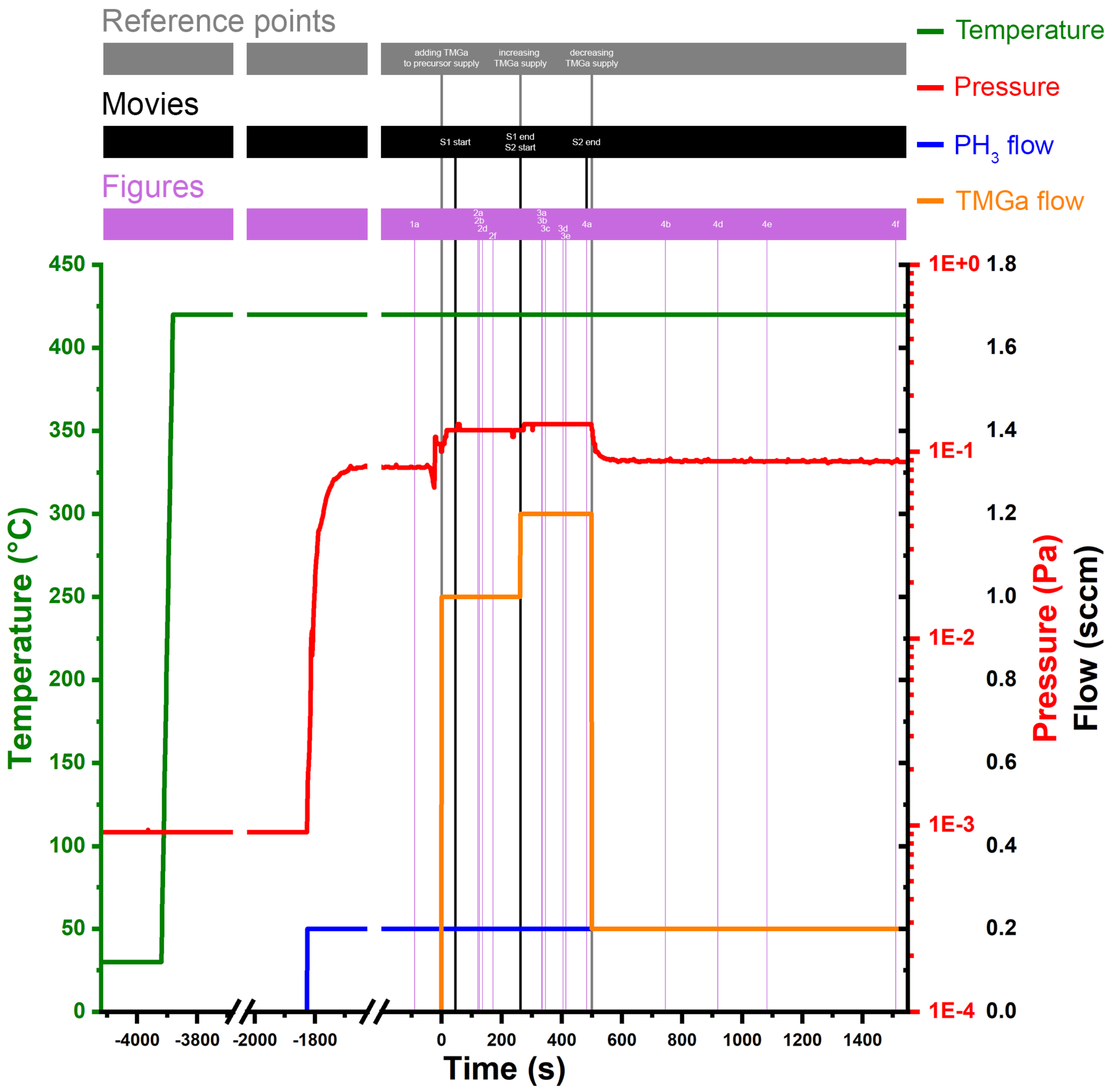

Figure S4. The graph reveals the variation of process conditions over time and specific experiment events. The curves in the graph include temperature (green line), column pressure (red line), PH_3 flow (blue line), and TMGa flow (orange line). The sample temperature was increased to 420 °C (timestamp: -3919 s), and subsequently, PH_3 was supplied to the sample (timestamp: -1825 s) until the end of the experiment (from −4120 s to 1550 s). The grey vertical lines indicate specific events crucial for the experiment, including adding TMGa to the PH_3 supply (timestamp: 0 s), increasing the TMGa supply (timestamp: 262 s), and decreasing the TMGa supply (timestamp: 499 s). Moreover, the events of acquiring HRTEM movies and images illustrated in figures of the main manuscript and movies in the *Supporting Information* are highlighted by purple and black vertical lines.

Table S1. Acquisition parameters for HRTEM images.

HRTEM image	Electron dose rate (e/Å2 s)	Exposure time (s)
Figure 1a	~3100	1.0
Figures 4b-c and S2a	~4200	1.0
Figure 4d	~4200	1.0
Figure 4e	~4400	1.0
Figure 4f and S2b	~4400	1.0

Table S2. Acquisition parameters for HRTEM movies.

HRTEM movie	Electron dose rate (e/Å2 s)	Frame rate (frames/s)
Movie S1	~2600	20
Movie S2	~1500	20

Table S3. Process parameters for the formation of Ag-Cu_3P nanoparticles by Ag-Cu seed particles.[3]

Figures	Movies	Temperature (°C)	Estimated PH_3 partial pressure at sample (Pa)	Estimated TMGa partial pressure at sample (Pa)	Estimated V/III ratio at the sample ( )	Estimated total pressure at sample (Pa)
1	-	420	$1.09 \cdot 10^{-1}$	-	-	$1.09 \cdot 10^{-1}$

Table S4. Process parameters for the GaP nucleation by Ag-Cu_3P seed particles.[3]

Figures	Movies	Temperature (°C)	Estimated PH_3 partial pressure at sample (Pa)	Estimated TMGa partial pressure at sample (Pa)	Estimated V/III ratio at the sample ( )	Estimated total pressure at sample (Pa)
2	S1	420	$1.29 \cdot 10^{-1}$	$3.57 \cdot 10^{-4}$	362	$5.87 \cdot 10^{-1}$
3 and 4a	S2	420	$1.22 \cdot 10^{-1}$	$4.06 \cdot 10^{-4}$	302	$6.42 \cdot 10^{-1}$

Table S5. Process parameters for the GaP growth facilitated by Ag-Cu_3P seed particles.[3]

Figures	Movies	Temperature (°C)	Estimated PH_3 partial pressure at sample (Pa)	Estimated TMGa partial pressure at sample (Pa)	Estimated V/III ratio at the sample ( )	Estimated total pressure at sample (Pa)
4b-f	-	420	$1.79 \cdot 10^{-1}$	$9.88 \cdot 10^{-5}$	1812	$3.06 \cdot 10^{-1}$

Table S6. Parameters for simulations of electron diffraction patterns.

Instrument: voltage	300 kV
Instrument: convergence	1°
Detector: spot size	0.02 $Å^{-1}$
Detector: saturation	100
Detector: gamma	2
Sample: thickness	100 Å
Sample: vol. fraction	100.0 %

Table S7. Processing parameters for extracting HRTEM images from the HRTEM movie raw data.

HRTEM image	Combine frames: count	Combine frames: method	Exposure time per frame (s)
Figure 2a	20	Average	0.05
Figures 2b-c	20	Average	0.05
Figures 2d-e	20	Average	0.05
Figure 2f	20	Average	0.05
Figure 3a	20	Average	0.05
Figure 3b	20	Average	0.05
Figure 3c	20	Average	0.05
Figure 3d	20	Average	0.05
Figure 3e	20	Average	0.05
Figure 4a	20	Average	0.05
Figures S3a-b	20	Average	0.05
Figure S3c	20	Average	0.05

Table S8. Processing parameters for extracting HRTEM movies from the HRTEM movie raw data.

HRTEM movie	Binning	Combine frames: count	Combine frames: skip	Combine frames: method	Processing: align/drift correction	Processing: alignment filter	Exposure time per frame (s)
Movie S1	4	20	20	Average	No	-	0.05
Movie S2	4	20	20	Average	No	-	0.05

References

1. Weibke, F.; Meisel, K.; Wiegels, L., Das zustandsdiagramm des systems silber-gallium. *Z. Anorg. Allg. Chem.* **1936,** *226* (2), 201-208.
2. Halder, S. K.; Sen Gupta, S. P., An X-ray determination of the thermal expansion of α-phase Ag–Ga alloys at high temperatures. *Acta Crystallogr. A* **1974,** *30* (6), 844-845.
3. Tornberg, M.; Maliakkal, C. B.; Jacobsson, D.; Wallenberg, R.; Dick, K. A., Enabling in situ studies of metal-organic chemical vapor deposition in a transmission electron microscope. *Microsc. Microanal.* **2022,** *28* (5), 1484-1492.
